# Plant resistance: scientific basis and latest research progress

**DOI:** 10.3389/fpls.2026.1789793

**Published:** 2026-04-01

**Authors:** Sunil Kumaraswamy, Yinghua Huang

**Affiliations:** 1 Department of Biology, Oklahoma State University, Stillwater, OK, United States; 2 Plant Science Research Laboratory, United States Department of Agriculture-Agricultural Research Service (USDA-ARS), Stillwater, OK, United States

**Keywords:** biotic stress, defense mechanisms, disease resistance, host plant resistance, insect pests, integrated pest management, plant protection, sustainable agriculture

## Abstract

Plant resistance to insects and diseases is a cornerstone of sustainable agriculture, reducing dependence on chemical pesticides and enhancing long-term crop resilience. Plant resistance is a suite of constitutive and inducible defenses, including structural barriers, biochemical defenses, signaling pathways activated upon recognition of pest or pathogen derived cues. Understanding how plants perceive biotic stress and mobilize these defenses through secondary metabolite production, reinforcement of physical barriers, and coordinated regulation of defense genes, is essential for designing effective management strategies Host plant resistance to insect herbivores exemplifies how specific plant traits can deter feeding, limit pest survival, or reduce reproduction. Advances in biotechnology, such as CRISPR/Cas9-based gene editing, RNA interference (RNAi), and transgenic approaches, have accelerated the development of crops with enhanced and durable resistance. These technologies enable precise manipulation of key resistance genes and pathways. Likewise, the integration of traditional methods with marker-assisted selection and genomic selection is improving the efficiency and accuracy of developing resistant cultivars. This review highlights the importance of dissecting plant-insect and plant-pathogen interactions at the molecular, biochemical, physiological levels to inform robust resistance integration. Future research that leverages advanced technologies and integrates resistance traits with agronomic performance will be pivotal for advancing sustainable pest management and ensure global food security. Together, these insights underscore the essential role of plant resistance in integrated pest management and crop improvement programs.

## 1 Introduction

Plants are continually exposed to diverse biotic and abiotic stresses and rely on multiple defense strategies to avoid, tolerate, or recover from such challenges (Hanley et al., 2007). Various organisms, including insects, pathogens, nematodes, and weeds, utilize plants as resources, but insect pests and microbial pathogens often cause substantial reductions in plant health, growth, and yield. Host plants, along with their nutritional status and metabolic composition, strongly influence insect population dynamics and the severity of plant disease. Over millions of years of evolution, plants have developed an extensive repertoire of protein-based defenses against biotic threats such as insects, fungi, and bacteria (Gimenez et al., 2018; Jha and Mohamed, 2022). Plant proteins and secondary metabolites function as enzyme inhibitors, toxins, or signaling molecules that disrupt pest physiology and development, forming a critical component of plant innate defense (Hussain et al., 2011; Jha and Mohamed, 2022).

Plants deploy three primary resistance mechanisms against insect pests: antixenosis, antibiosis, and tolerance. Antixenosis (non-preference) encompasses traits that affect pest behavior, reducing settling, feeding or oviposition (Painter, 1951). Antibiosis directly alters pest biology, reducing growth, survival, fecundity, and lifespan (Painter, 1951; Smith, 2005). Tolerance allows plants to endure or recover from pest damage while maintaining productivity (Panda and Khush, 1995). Incorporating antixenosis and antibiosis traits into crop breeding is essential for sustainable, cost-effective pest management (Kumaraswamy et al., 2024b). Physical traits such as trichomes, leaf toughness, and cuticular thickness, serve as direct defenses, limiting herbivore feeding and development (Kumaraswamy et al., 2024b; Samadia and Haldhar, 2019). These traits can reduce pest use of plants for feeding, oviposition, or shelter, thereby influencing host preference through antixenosis (War et al., 2012). In combination, antibiosis, antixenosis, and tolerance shape plant-insect interactions (Iqbal et al., 2018; Koch et al., 2016), with antibiosis disrupting pest physiology, antixenosis, altering pest behavior, and tolerance mitigating the consequences of injury.

Host plant resistance (HPR) is a cornerstone of pest management and remains one of the most effective and environmentally sustainable agricultural strategies (Horgan et al., 2020; Mansour and Eryan, 2022). Crops with inherent resistance traits sustain less damage, underscoring the importance of breeding programs that integrate HPR mechanisms. These strategies have been widely and successfully applied against insect pests (Haldhar et al., 2015; War et al., 2012). Leveraging wild gene pools to develop stress-resistant genotypes, together with conservation of related species, offers significant opportunities for future crop improvement (Kumaraswamy et al., 2025; Samadia and Haldhar, 2017). Resistant cultivars continue to serve as fundamental components of integrated pest management (IPM), providing effective protection against insect pests and vector-borne diseases (Huang, 2011).

Despite these advances, pest management in many cropping systems still depends heavily on chemical inputs, raising environmental and health concerns. As a result, agriculture is increasingly adopting biotechnology to enhance crop resilience, productivity, and sustainability (Dara, 2019; Nawaz et al., 2019). Genetic engineering and modern molecular tools have significantly advanced pest management, evolving from early *in vitro* experiments to impactful field applications. Microbial technologies also offer promising alternatives (Kamthan et al., 2016), exemplified by the success of *Bacillus thuringiensis* (Bt) genes in protecting crops from insect damage (Khan et al., 2012). Emerging biotechnologies mark a new era of precision pest management. Traditional genetic modification (GM) enabled crops with improved traits such as pest resistance and enhanced nutrition. Today, RNA interference (RNAi) and CRISPR/Cas9 gene editing offer even more targeted approaches. RNAi can specifically silence pest or pathogen genes, providing an eco-friendly alternative to chemical pesticides (Adeyinka et al., 2020; Mehlhorn et al., 2021). CRISPR/Cas9 enables precise genome edits to enhance plant resistance and engineer targeted control of insect pests (Perkin et al., 2016; Singh et al., 2022).

Integrating emerging biotechnologies into agriculture has the potential to reduce reliance on chemical pesticides, enhance climate resilience, conserve beneficial biodiversity, and strengthen global food security. Although synthetic pesticides have historically contributed to pest suppression and yield improvement, their widespread use has also caused ecological damage and posed risk to human health, natural ecosystems and biodiversity (Aktar et al., 2009). HPR offers a natural, sustainable alternative, grounded in evolutionary defense mechanisms (Sharma and Ortiz, 2002). By incorporating HPR into crop improvement programs, farmers can lessen pesticide inputs and their environmental footprint. Moreover, HPR remains effective against a broad range of pests, including those that have developed resistance to chemical insecticides, because it is a dynamic system capable of responding to evolving pest pressures (Huot et al., 2013). Thus, HPR provides a long-term, environmentally sound strategy for safeguarding agricultural productivity.

## 2 Types of plant resistance

### 2.1 Constitutive resistance

**Figure 1:**
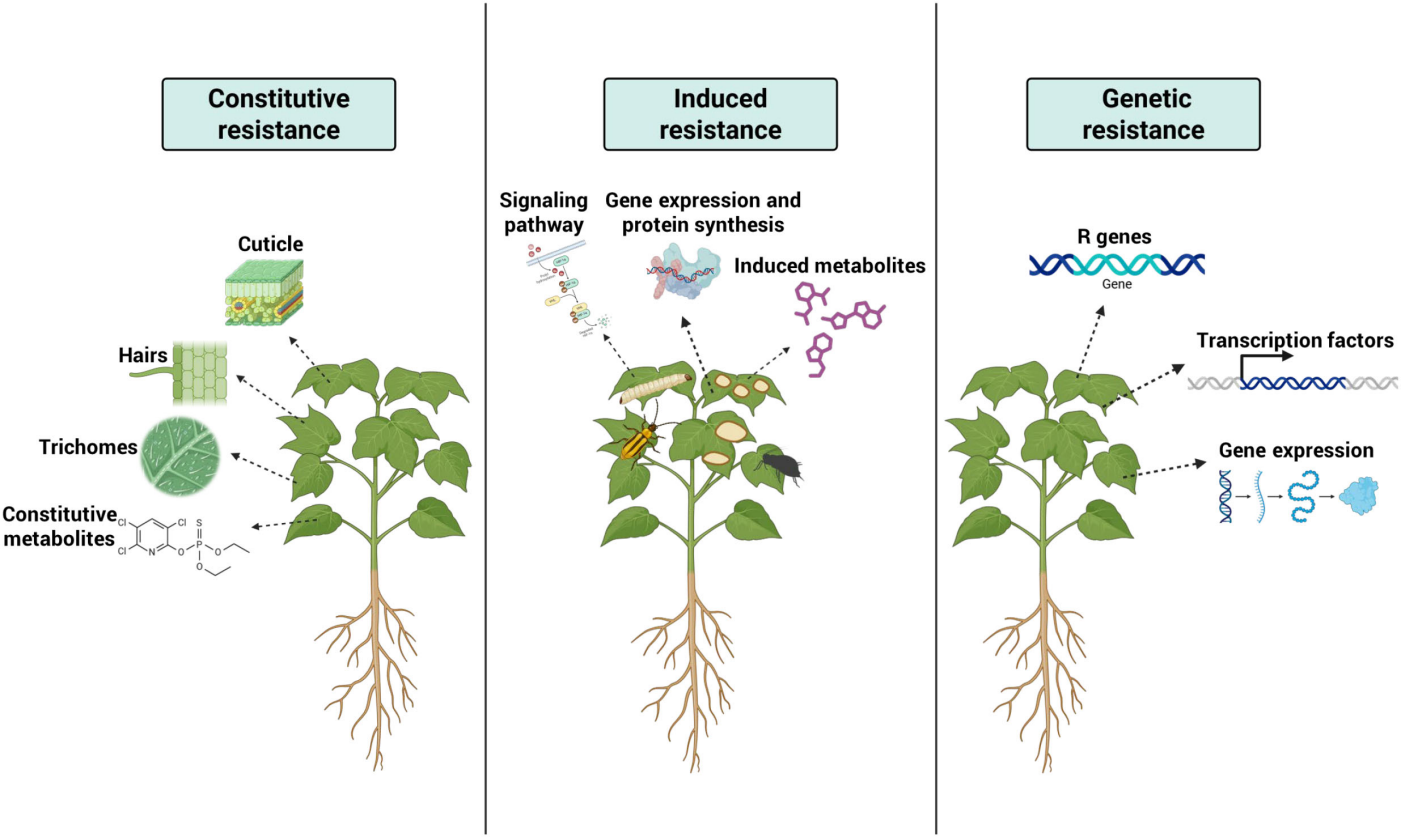
Illustration of types of plant resistance to insect pests and diseases.

Plants defend themselves against pests and pathogens through constitutive and induced mechanisms. Constitutive resistance encompasses preformed defenses that function independently of herbivore or pathogen attack (Bar and Shtein, 2019). These include physical barriers such as hairs, trichomes, thorns, spines, and thicker leaves (Samadia and Haldhar, 2019) (**Figure 1**). Structural and chemical defenses may act independently or in combination, for instance, glandular trichomes and their secretory canals from an integrated protective system (Glas et al., 2012; Wang et al., 2021). Constitutive chemical defenses may be toxic, deterrent, or antifeedant, reducing survival and fecundity (Silva et al., 2019; War et al., 2012). Additional examples include thick cell walls, cutin and suberin layers, antimicrobial peptides, and secondary metabolites such as phenols, saponins, and glucosinolates (War et al., 2012). Constitutive resistance is often associated with species adapted to stressful environments, where slow growth and constant investment in defense enhance survival. At the molecular level, constitutive resistance may involve resistance (R) genes that are continuously active; in some cases, mutations result in the persistent expression of defense responses even in the absence of pathogens (Zhang et al., 2003).

### 2.2 Induced resistance

Induced defenses are activated in response to biotic or abiotic stress and involve structural modification, production of secondary metabolites, and the synthesis or upregulation of defense-related proteins following attack (Kersch-Becker et al., 2019). These responses are coordinated by complex phytohormone signaling networks, primarily involving salicylic acid (SA), jasmonic acid (JA), and ethylene (ET) (Howe et al., 2018; Schuman and Baldwin, 2016) (**Figure 1**). Induced responses may be localized or systemic, including systemic acquired resistance (SAR) or induced systemic resistance (ISR). These defense pathways are characterized by the accumulation of pathogenesis-related (PR) proteins, antimicrobial compounds, and reinforcement of cell walls. In the rhizosphere, beneficial microbes like *Bacillus* and *Pseudomonas* species produce elicitor-active molecules that stimulate ISR (Choudhary et al., 2007; War et al., 2012). Plant responses to elicitors are strongly influenced by genotypic variation, with cultivars of the same species often exhibiting markedly different levels of defense activation (Bruce, 2014).

Elicitors commonly function through priming, a mechanism that prepares plants to mount faster and stronger immune responses upon subsequent attack. However, the effectiveness of priming varies considerably among genotypes (Giovannoni et al., 2021; Twamley, 2025). Therefore, selection of elicitor responsive genotypes is crucial. Induced resistance can reduce the performance of later-attacking pests and pathogens following prior herbivory or infection (Abo-Elyousr et al., 2008). Herbivory also alters plant metabolomic profiles to limit invasion (Jiang et al., 2019; Kumaraswamy et al., 2025) and stimulates the emission of volatile organic compounds (VOCs) that attract natural enemies of herbivores. Plants perceive damage-associated molecular patterns (DAMPs), herbivore-associated molecular patterns (HAMPs) and pathogen-associated or microbe-associated molecular patterns (PAMPs/MAMPs) which activate layered defense responses (Quintana-Rodriguez et al., 2018; Santamaria et al., 2018; Yu et al., 2024). Both constitutive and induced defenses may operate directly through toxins or structural barriers that impair herbivore growth (Lin et al., 2020; Yang et al., 2020) or indirectly by releasing signaling molecules that recruit predators or alert neighboring plants (Coppola et al., 2017).

### 2.3 Genetic resistance

Genetic resistance to biotic stress stems from molecular mechanisms that enable rapid pathogen perception and activation of defense responses. Central to this process are R genes, which recognize pathogen-derived effectors and initiate defense signaling cascades that can trigger the Hypersensitive Response (HR) and subsequently activate SAR (Purayannur et al., 2017) (**Figure 1**). Most R genes encode proteins containing conserved domains such as nucleotide-binding sites (NBS) and leucine-rich repeats (LRR), which mediate effector recognition and downstream signaling through phosphorylation events and nucleocytoplasmic trafficking (Wu et al., 2014; Chen et al., 2023). These interactions often lead to the accumulation of PR proteins and strengthen immune responses (Hammond‐Kosack and Kanyuka, 2007). R genes frequently occur in genomic clusters and evolve rapidly under positive selection, particularly within LRR regions, enabling adaptation to continuously evolving pathogens (Hammond‐Kosack and Kanyuka, 2007). In crop improvement programs, advanced biotechnological approaches such as gene editing and R-gene stacking are used to develop durable, broad-spectrum resistance in major crops including rice, wheat, and potato (Simon et al., 2023; Rubiales et al., 2023). R gene-mediated resistance is further integrated with phytohormone signaling networks involving SA, JA, ET, and abscisic acid (ABA) (Li et al., 2019).

Transcription factors (TFs) act as master regulators of defense gene expression orchestrating large-scale transcriptional reprogramming during biotic stress. Prominent TF families including WRKY, NAC, MYB, and bZIP play critical roles in modulating immune responses (Meraj et al., 2020; Zhang and Huang, 2024). WRKY TFs bind W-box elements to regulate SA- and JA-dependent pathways, functioning as either activators or repressors that influence programmed cell death and secondary metabolite biosynthesis (Li et al., 2025b; Shrestha et al., 2024; Wang et al., 2025). NAC TFs coordinate defense-related transcriptional reprogramming by activating defense genes or hormone signaling components (Zhang and Huang, 2024). While some NAC members enhance HR-associated cell death as positive regulators, others function as negative regulators; their activity is often fine-tuned by microRNAs and post-translational modifications (Bian et al., 2020; Nuruzzaman et al., 2013). MYB TFs contribute to growth-defense balance by regulating secondary metabolism and modulating hormone (SA, JA, ET) signaling during pathogen attacks (Imran et al., 2025). bZIP TFs are integral to hormone-mediated defense, particularly through interactions with NPR1 and TGA factors in SA-dependent pathways. Studies in species such as soybean and *Arabidopsis* demonstrate that bZIP proteins enhance pathogen resistance while also participating in abiotic stress adaptation (He et al., 2020; Llorca et al., 2014). Collectively, TFs integrate hormonal signaling (SA, JA, ET, ABA), reactive oxygen species (ROS) dynamics (Pant and Huang, 2020), MAP kinase cascades, and the ubiquitin-proteasome system to fine tune immune outputs. Through coordinated regulation of PR gene expression, metabolite biosynthesis, and programmed cell death, TF networks ensure a balanced and effective defense response (Burke et al., 2020; Liu et al., 2023).

## 3 Mechanisms of plant resistance

### 3.1 Innate immune system

**Figure 2:**
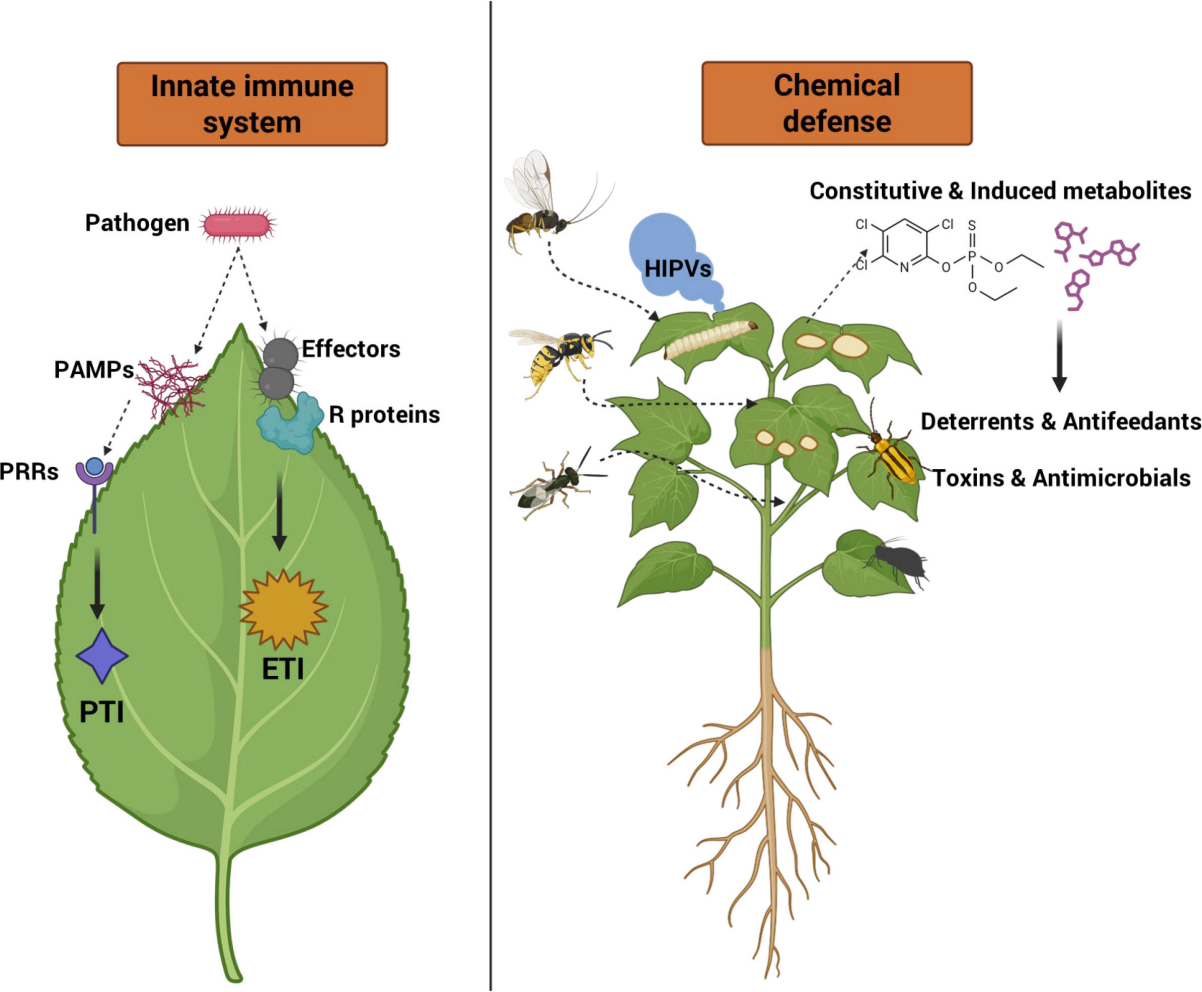
Illustration of mechanisms of plant resistance to insect pests and diseases.

Plants have evolved a sophisticated innate immune system to cope with stresses imposed by pathogens and herbivores (Miller et al., 2016). Constant exposure to diverse microorganisms requires plants to distinguish self from non-self and mount appropriate responses through tightly interconnected signaling networks (Saijo and Loo, 2019). This immune system integrates constitutive and inducible defenses, mediated by cellular receptors that activate downstream pathways to restrict pathogen invasion and herbivore colonization (Schulze et al., 2018). Plant immunity operates through a two-tier defense system that recognizes conserved microbial signature and pathogen-derived effectors to initiate protective responses (Jones and Dangl, 2006) (**Figure 2**). Discoveries of resistance genes and immune receptors have enabled modern resistance breeding and provide a foundation for improving durable crop protection under evolving environmental challenges (Jones et al., 2024). The first layer, pattern-triggered immunity (PTI), offers broad-spectrum resistance and is activated when cell-surface pattern-recognition receptors (PRRs), such as receptor-like kinases, detect conserved DAMPs, HAMPs and PAMPs/MAMPs (Quintana-Rodriguez et al., 2018; Santamaria et al., 2018; Yu et al., 2024). Because these molecular signatures are essential for pathogen survival, they are relatively conserved and less prone to mutation (Yu et al., 2024). Activation of PRRs rapidly induces ion fluxes, ROS production, and MAPK kinase cascades, followed by callose deposition and large-scale transcriptional reprogramming (Weralupitiya et al., 2024). To overcome PTI, pathogens deploy virulence factors that suppress host defense. In response, resistant plants with R genes activate the second layer, effector-triggered immunity (ETI), a more robust and sustained defense mechanism mediated primarily by resistance (R) proteins of the nucleotide-binding site-leucine-rich repeat (NBS-LRR) family (Muthamilarasan and Prasad, 2013; Wu et al., 2014) (**Figure 2**). These intracellular receptors recognize specific pathogen effectors and initiate strong defense signaling. ETI often culminates in a HR, a localized form of programmed cell death that restricts pathogen spread (Muthamilarasan and Prasad, 2013). This process involves extensive transcriptional reprogramming, the integration of kinase cascades, phytohormonal signaling, and transcription factor networks to coordinate an effective immune defense (Bentham et al., 2020).

### 3.2 Chemical defense

#### 3.2.1 Plant metabolites in resistance

Chemical defenses, both constitutive and inducible, are central to plant resistance against insect pests and pathogens (Shakeel et al., 2025; Upadhyay et al., 2024) (**Figure 2**). Plants synthesize a diverse array of bioactive metabolites, including toxins, repellents, and antifeedants, many of which serve as eco-friendly alternatives to synthetic pesticides (Anjali et al., 2023; Shakeel et al., 2025). Among these, secondary metabolites such as flavonoids, terpenoids, phenolics, and alkaloids play critical roles in plant-insect interactions. Herbivore attack frequently induces accumulation of these compounds, thereby impairing insect growth, survival, and reproduction (Divekar et al., 2022; Jeckel et al., 2022). Their defensive functions include direct toxicity, feeding deterrence, and the emission of protective volatiles (Divekar et al., 2022). Induced resistance is often accompanied by increased production of VOCs, fatty acids, and phenylpropanoids that deter insects or recruit natural enemies (Kumaraswamy et al., 2025; Roumani et al., 2022). Specific metabolites, such as galactaric acid and hydroxycinnamic acids provide resistance against whitefly-mediated viruses, while phenolic compound can inhibit insect digestive enzymes (Lu and Xia, 2025; Rossouw et al., 2019). These chemical defenses are tightly regulated through phytohormone crosstalk, primarily involving JA, ET, and SA, which prioritize responses according to the nature of attackers (Bonaventure, 2012; Smith and Boyko, 2006; Huang et al., 2022). For instance, spider mite infestation activates JA and SA mediated signaling in pepper, while SA-dependent SAR and the marker gene *PR1b1* contribute to resistance in eggplant (Chen et al., 2021; Zhang et al., 2020a).

Defense-related proteins further enhance chemical resistance. Protease inhibitors and peroxidases suppress insect development and digestion (Wang et al., 2018; War et al., 2012). To activate these pathways, plants differentiate mechanical damage from insect feeding by recognizing elicitors present in oral secretions, which initiate signaling cascades and ROS (Bonaventure, 2018; Villarroel et al., 2016). Although excessive ROS can cause cellular damage, controlled ROS bursts function as key signal molecules that drive death, they also act as messengers for metabolic reprogramming (Rashid and Chung, 2017; Pant and Huang, 2020; Wani et al., 2022). In defense against pathogens, SA-regulated PR proteins, antimicrobial phytoalexins, and structural reinforcements such as lignification collectively restrict infection (Guerreiro and Marhavý, 2023; Hönig et al., 2023). Additionally, metabolite classes such as saponins and terpenoids offer broad-spectrum antifungal and nematocidal protection (Shakeel et al., 2025).

#### 3.2.2 Plant volatiles in resistance

When attacked by herbivores, plants release distinct blends of herbivore-induced plant volatiles (HIPVs), including terpenoids and fatty acid derivatives that differ markedly from their constitutive emissions (Bouwmeester et al., 2019; Pérez-Hedo et al., 2021). These low-molecular-weight compounds function primarily as indirect defenses by deterring insects, attracting species-specific natural enemies, and priming resistance in neighboring plants (Silva et al., 2017; Zhou and Jander, 2022). For example, volatiles emitted from herbivore-infested potato plants enhance resistance in nearby plants against *Spodoptera exigua* (Vázquez-González et al., 2023). Beyond ecological signaling, VOCs can stabilize cellular membranes, scavenge ROS, and contribute to the establishment of systemic resistance (MacDougall et al., 2022; Russo et al., 2022). In tomato and pepper plants, herbivore infestation elevates volatile emissions that disrupt whitefly behavior and population dynamics (Ghosh et al., 2022), while exposure to exogenous cues such as (Z)-3-hexenol can prime JA-dependent defense signaling (Su et al., 2020). Exploiting such compounds, including the repellent terpene 7-epizingiberene or methyl salicylate, offers promising, eco-friendly strategies for managing insect vectors such as *Bemisia tabaci* (Rosen et al., 2015; Shi et al., 2016). In addition, plants can metabolically convert perceived airborne signals, such as (Z)-3-hexenyl propanoate, into more toxic defense compounds that reduce susceptibility to pests like *Phthorimaea absoluta* (Pérez-Hedo et al., 2021).

#### 3.2.3 Tri-trophic interactions in resistance

HIPVs are therefore central to indirect defense, serving as reliable cues that guide natural enemies to herbivore-infested plants while simultaneously repelling pests (Ayelo et al., 2021; Turlings and Erb, 2018). For example, infestation by *Myzus persicae* induces volatile emission in pepper and cabbage that attract the parasitoid *Aphelinus varipes* (Ali et al., 2022), whereas *Macrosiphum euphorbiae* infestation in tomato plants recruits *Aphidius ervi* (Coppola et al., 2019). Similarly, *P. absoluta* feeding stimulates monoterpenes release that attracts the predator *Nesidiocoris tenuis* (Ayelo et al., 2021), and volatiles from cucumber and potato plants guide the ant *Formica pratensis* (Schettino et al., 2017). Beyond herbivory, predator activity itself can amplify plant defense, for example, feeding by *N. tenuis* primes JA and ABA pathways, deterring pests such as *B. tabaci* while recruiting of parasitoids like *Encarsia formosa* (Pérez-Hedo et al., 2015). These volatile-mediated defenses operate both locally and systemically; for example, *N. tenuis*-induced compounds like (Z)-3-hexenyl propanoate can prime neighboring undamaged plants for enhanced resistance (Pérez-Hedo et al., 2017, 2021).

## 4 Host plant resistance to insect herbivores

HPR refers to genetically based plant traits that reduce herbivore survival, development, or reproduction through genetic traits. HPR can be categorized into three main types: antixenosis (non-preference), where plants deter insect colonization or feeding; antibiosis, traits that negatively affects insect biology after feeding; and tolerance, where plants’ ability to withstand injury without significant yield loss. These mechanisms involve morphological traits (e.g., trichomes, wax layers), biochemical defenses (e.g., secondary metabolites, proteinase inhibitors), and induced signaling pathways (via JA and SA) (Shrestha et al., 2024). HPR is a sustainable and environmentally friendly pest management strategy, reducing reliance on chemical pesticides and contributing to IPM programs.

### 4.1 Antixenosis

**Figure 3:**
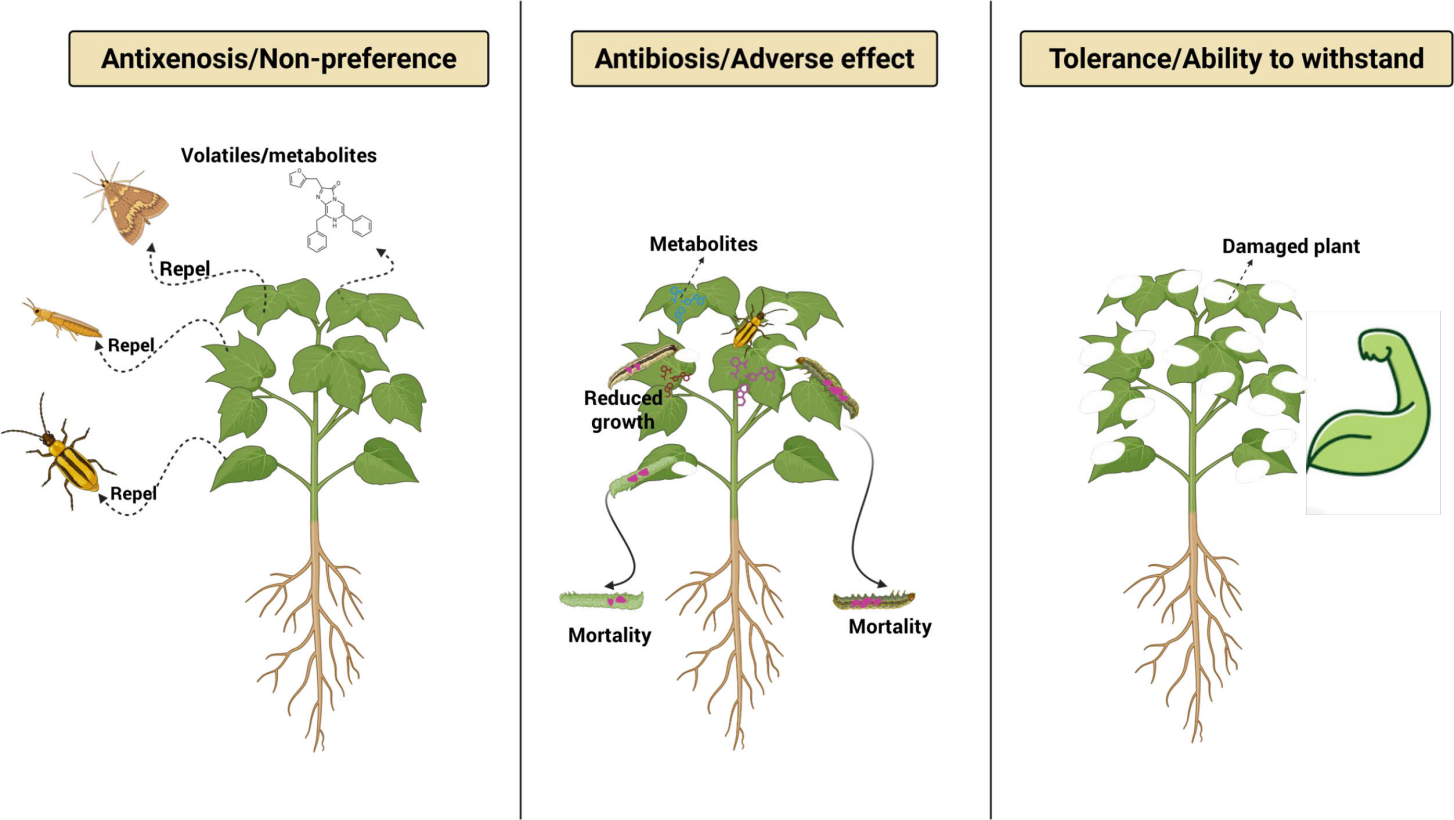
Illustration of Host plant resistance (HPR) mechanisms to insect pests.

Antixenosis, or “non-preference,” involves plant traits that deter insects from feeding, oviposition, or sheltering, thereby reducing infestation levels (Kogan and Ortman, 1978) (**Figure 3**). Acting in concert with antibiosis and tolerance, antixenosis relies on physical and chemical characteristics that modify insect behavior before substantial damage occurs (Kogan and Ortman, 1978). Physical defenses include leaf toughness, surface waxes, coloration, and tissue thickness, all of which can influence host selection (Xing et al., 2017). Trichomes play a particularly important role: non-glandular trichomes can hinder insect movement and reduce pest longevity, as observed in kidney bean (Xing et al., 2017), while glandular trichomes secrete sticky or toxic exudates that deter spider mites and whiteflies in cucumber (Wheeler and Krimmel, 2015; Xue et al., 2019). Chemical antixenosis involves the production of deterrent, toxic, or unpalatable secondary metabolites. For instance, glucosinolates in *Brassica* species repel weevils and other herbivores (Ellis and Farrell, 1995; Tansey et al., 2010). Resistance may also occur at the phloem level; in wild potato species, specific resistance loci interfere with aphid settling and sustained feeding (Klingler et al., 2005; Roux et al., 2008). In soybean, high trichome density and greater leaf thickness reduce infestation by *Helicoverpa armigera* and whiteflies (Ongaratto et al., 2021). Similarly, resistant rice and tomato genotypes employ structural and chemical cues to limit oviposition and colonization by planthoppers, gall midges, and fruit borers (Prahalada et al., 2017; Sahu et al., 2022). Likewise, maize cultivars also utilize comparable morphological and chemical traits to minimize pest damage (Castro et al., 1999).

### 4.2 Antibiosis

Antibiosis is a resistance mechanism in which plant traits directly impair insect herbivore growth, reproduction, or survival after feeding (Painter, 1951) (**Figure 3**). This form of resistance arises from biochemical and physiological alterations, including the accumulation of toxic secondary metabolites, modification of nutrient composition, or production of defensive proteins, that create a hostile internal environment for herbivores (Bischoff et al., 2023; Painter, 1951; Rashid and Chung, 2017). These defensive responses are frequently regulated by JA and ET signaling pathways (Rashid and Chung, 2017). By extending developmental time, reducing fecundity, and increasing mortality, antibiosis can limit insect biomass and population growth (Painter, 1951). In wheat, specific resistance genes confer antibiosis against greenbugs and Russian wheat aphids, by reducing insect longevity and developmental rates (Castro et al., 1999). Similarly, in rice the *Bph31* gene mapped to the long arm of chromosome 3 from cultivar ‘CR2711-76’, confers resistance to brown planthopper (BPH) through combined resistance mechanisms (Panda and Heinrichs, 1983; Prahalada et al., 2017). Introgression of *Bph31* gene into *indica* rice variety “Jaya” through Marker-assisted selection (MAS) significantly enhances resistance to multiple BPH biotypes (Sriram et al., 2024). While *Bph1* confers antibiosis and tolerance, offering moderate BPH resistance, *Bph31* offers antixenosis and tolerance, strengthening overall defense against BPH (Huang et al., 2025). Comparable antibiosis effects have been reported in *Gossypium* species against whiteflies (Jindal et al., 2007) and in resistant chickpea genotypes against *H. armigera* (Golla et al., 2018). Defensive proteins further exemplify antibiosis. Protease inhibitors and toxic proteins disrupt insect digestion and nutrient assimilation (Casaretto and Corcuera, 1995). For instance, soybean’s Bowman-Birk inhibitor impairs insect pests such as corn earworm (Sultana et al., 2023), while lectins derived from beans exhibit toxicity toward aphids and caterpillars (Li et al., 2023b).

### 4.3 Tolerance

Plant tolerance is a form of resistance in which plants maintain growth, reproduction, and overall fitness despite insect injury (**Figure 3**). Unlike antibiosis or antixenosis, tolerance does not directly affect insect survival or behavior; instead, it reduces the fitness consequences of injury, making it a durable, sustainable defense mechanism (Peterson et al., 2017). Tolerance is supported by multiple physiological adjustments. These include delayed phenological development, compensatory growth, and enhanced photosynthetic capacity driven by increased levels of chlorophyll a, chlorophyll b, and carotenoids and carbon assimilation enzymes (Stenberg and Muola, 2017; Weintraub et al., 2018). Plants may also mobilize stored assimilates to damaged tissues and elevate oxidative enzymes such as peroxidases to mitigate stress and maintain cellular homeostasis (Koch et al., 2016; Strauss et al., 2003). Morphological plasticity further contributes to tolerance, with plants producing additional branches, tillers, or roots and adjusting root-to-shoot ratios to compensate for lost biomass (Koch et al., 2016; Strauss et al., 2003). Tolerance capacity often increases with plant age, as mature plants typically possess greater resource reserves and improved acquisition efficiency (Gols, 2024; Hoque and Avila-Sakar, 2014). Because tolerance is generally polygenic and genetically distinct from antibiosis and antixenosis it represents a key target in breeding programs aimed at sustainable pest management breeding (Ferrero et al., 2019; Wani et al., 2022).

### 4.4 Pseudo resistance (host evasion and escape)

Pseudo-resistance refers to environmentally influenced, non-genetic traits that reduce insect damage without conferring true physiological resistance (Beck, 1965). These strategies often rely on ecological or developmental factors that limit plant-insect interactions. For example, host escape and evasion occur when plants avoid peak pest pressure through spatial or temporal mismatches, such as completing vulnerable growth stages before herbivore populations reach damaging levels (Marquis and Moura, 2021; Stenberg and Muola, 2017). At the population level, synchronized phenology or habitat-mediated concealment can collectively reduce herbivore impact. Morphological and biochemical trait also contributes to pseudo-resistance (Wani et al., 2022). Plants may alter growth architecture and employ mimicry, such as producing egg-like structures or resin spots, to mislead herbivores and deter oviposition (Leather, 2017). Structural characteristics including increased leaf thickness or trichome density can make plants less detectable or accessible, while associate resistance occurs when neighboring vegetation reduces herbivore colonization (Dominguez et al., 2017). Additionally, VOCs may repel insects or prime defense responses without directly impairing herbivore performance (Conboy et al., 2020; Nalam et al., 2019; Piesik et al., 2022). Although pseudo-resistance does not suppress insect performance directly, it can substantially reduce damage and serve as a valuable complement to IPM strategies (Leather, 2017; War et al., 2012).

## 5 Recent advances in plant resistance research

**Figure 4:**
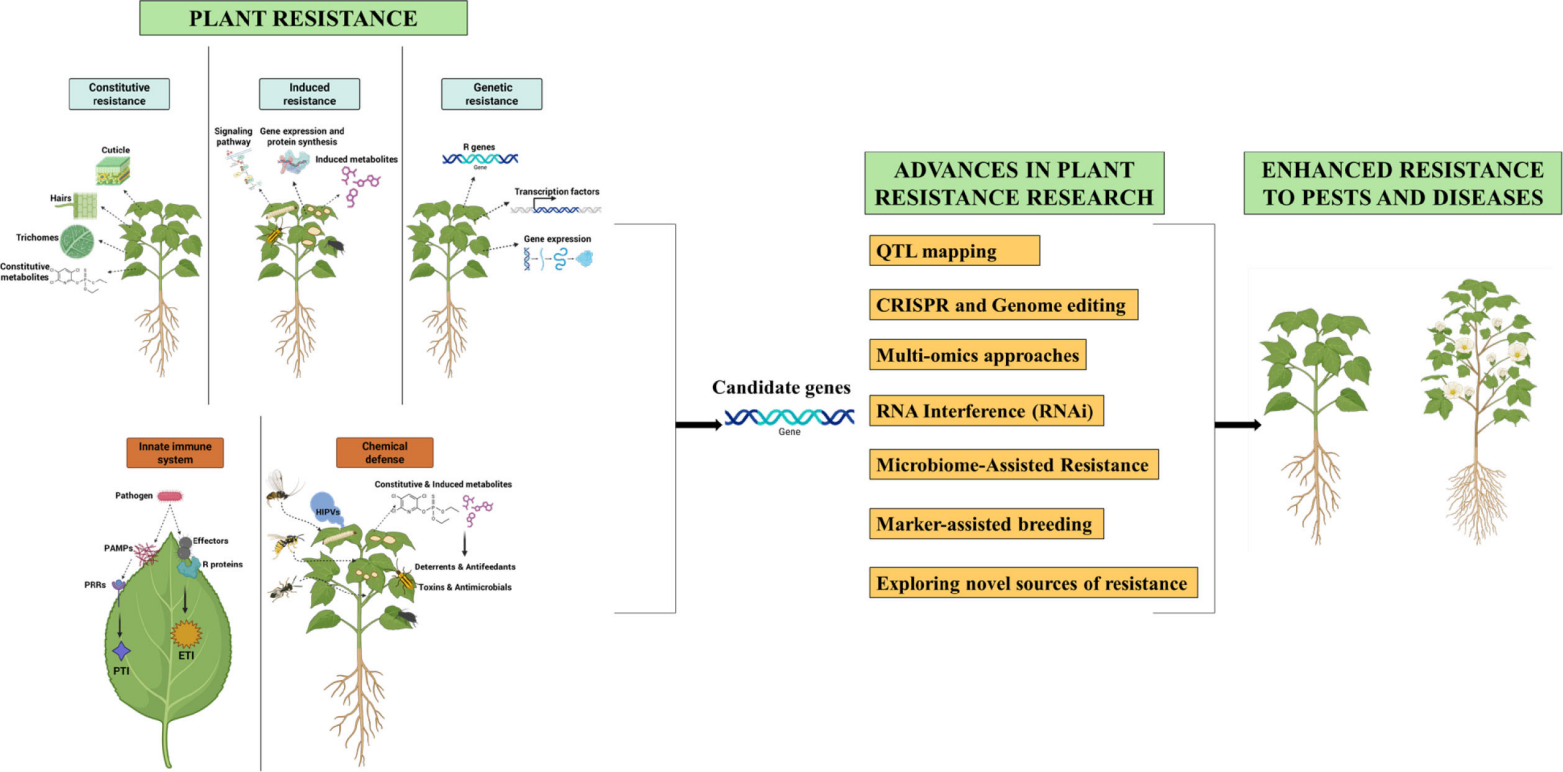
Illustration of utilization of plant resistance for the crop improvement.

Recent advances in plant resistance research have shifted from traditional breeding toward high-precision molecular approaches and genomics-assisted breeding. A significant breakthrough has been achieved through utilizing different plant resistance traits and by employing several advanced approaches to incorporate more durable, broad-spectrum protection in plants against insect pests and pathogens (**Figure 4**).

### 5.1 Quantitative trait locus mapping

QTL mapping is a fundamental approach for identifying genomic regions associated with plant resistance traits and has greatly advanced the development of insect and disease resistant crop varieties. The use of cutting-edge tools, such as single nucleotide polymorphism (SNP) markers and genotyping by sequencing (GBS), has increased mapping resolution and efficiency, even in crops with limited genomic resources. Because insects impose diverse forms of physiological, biochemical, and structural damage, QTL mapping is increasingly combined with other “omics” approaches such as transcriptomics, proteomics, and metabolomics to dissect resistance mechanisms at multiple biological levels. Wild relatives remain important reservoirs of resistance traits, such as glandular trichomes and defensive metabolites which can be identified as specific QTLs and introgressed into cultivated crops (Vosman et al., 2018). Significant QTLs have been identified for resistance to insects in different crops (Emebiri et al., 2016; Jiménez-Galindo et al., 2017; Vosman et al., 2018; Wu and Huang, 2008; Punnuri and Huang, 2017; Zhang and Huang, 2025). In tomato, for example, QTLs associated with trichome density and metabolite production overlap with loci, conferring whitefly resistance. In maize, QTLs have been linked to yield components and resistance to Mediterranean corn borer highlight the potential to simultaneously enhance productivity and pest resistance (Jiménez-Galindo et al., 2017; Vosman et al., 2018). Modern breeding techniques, including MAS and genome editing, now enable targeted exploitation of resistance QTLs, supporting the development of cultivars with durable insect resistance and reducing reliance on chemical controls (Wani et al., 2022; Zhang and Huang, 2025).

QTL mapping has also been instrumental in resolving genetic basis of quantitative disease resistance (QDR). Unlike qualitative resistance governed by single major genes, QRD is typically polygenic, race-nonspecific, and environmentally influenced. Resistance conferred by QTLs is typically quantitative, involving multiple genes with minor effects that often show environmental sensitivity. Molecular markers linked to quantitative resistance loci (QRLs) facilitate MAS, allowing efficient integration of disease resistance into breeding programs (StClair, 2010). QTLs contributing to QRD have been identified for rice blast, potato late blight, grey leaf spot in maize, bacterial wilt in tomato, and soybean cyst nematode, among other pathosystems (Young, 1996). This method provides insight into QDR, characterized by partial protection rather than immunity and often shaped by epistatic and environmental interactions. Advanced statistical approaches like mixed models and Bayesian inference improve mapping accuracy by accounting for genotype-by-environment interactions. Gene pyramiding through MAS or genetic engineering enhances durability and broad-spectrum resistance. Successful QTL mapping in crops such as wheat, maize, rice, and tomato has led to the identification of loci for resistance to Fusarium head blight, leaf rust, powdery mildew, sheath blight, and tomato spotted wilt virus, without negatively impacting traits like plant height or flowering time (Buerstmayr et al., 2009; Hu et al., 2022; Li et al., 2014; Pereira et al., 2000).

QTL mapping is a powerful approach for identifying genomic regions linked to resistance traits in plants, requiring precise phenotyping, robust statistical models, and well-structured mapping populations (Kulwal, 2018). Both biparental linkage and association mapping are used based on the species and genetic material available (Sehgal et al., 2016). Resistance traits often involve complex genetic architectures with multiple small-effect loci and genotype-by-environment interactions, posing challenges for breeding. Single-gene resistance may break down quickly due to pest or pathogen evolution, making QTL pyramiding essential for durable resistance. Accurate phenotyping, particularly for insect resistance, remains a bottleneck, but integration with high-throughput and multi-omics data offers promise. Overall, QTL mapping has advanced resistance breeding by enabling MAS and gene pyramiding, supporting the development of resilient crops (StClair, 2010; Wani et al., 2022; Young, 1996).

Genome-Wide Association Studies (GWAS) have become a powerful approach for dissecting the genetic architecture of plant resistance by exploiting natural genetic diversity and historical recombination to identify high resolution QTLs, SNPs, and candidate genes associated with plant defense (Gangurde et al., 2022; Xiang et al., 2024). GWAS has been widely applied to uncover loci associated with resistance to pests and diseases across major crops (Badji et al., 2020; Gangurde et al., 2022; Xiang et al., 2024). For instance, GWAS in rice identified the *OsRSSB4* gene as a positive regulator of resistance to striped stem borer (Xiang et al., 2024), while studies in maize revealed loci associated to resistance to fall armyworm and maize weevil (Badji et al., 2020). Similarly, GWAS in rice revealed candidate genes and QTLs associated with resistance to blast and blight diseases, whereas in wheat, GWAS has mapped resistance loci for stripe rust, leaf rust, and Fusarium head blight (Gangurde et al., 2022). Overall, the shift from traditional QTL mapping to high-resolution GWAS has significantly advanced the discovery of resistance loci and accelerated resistance breeding.

Marker-assisted breeding (MAB) has become a central strategy in resistance breeding by enabling efficient and precise selection of genotypes carrying desirable alleles (Anand et al., 2023; Onkarappa et al., 2024). Unlike conventional phenotypic selection, MAB relies on molecular markers linked to resistance loci to identify superior genotypes early in the breeding cycle (Miedaner and Korzun, 2012). This approach has facilitated pyramiding of multiple resistance genes, such as stacking green rice leafhopper resistance genes in rice (Jena and Mackill, 2008), and has been widely applied in crops including as sorghum, soybean, chickpea, mungbean, and cowpea to improve resistance to aphids, whiteflies, and legume pod borers (Onkarappa et al., 2024). In cassava, MAB has reduced breeding time by at least four years for resistance to cassava mosaic disease, green mite, and whitefly. In peanuts, nematode-resistance genes from wild relatives have been incorporated into new cultivars, while QTLs for resistance to aphids and tomato spotted wilt virus have been identified and deployed (Burow et al., 2013). Overall, MAB significantly accelerates the development of pest and disease resistant cultivars and enhances the efficiency of early generation selection.

MAB has also played a major role in disease resistance breeding. In rice, it has enabled the development of cultivars resistant to major diseases such as bacterial blight and blast. For example, “Improved Samba Mahsuri” (ISM) carries three bacterial blight resistance genes (*xa21, xa13, xa5*) and has been further improved through pyramiding of blast resistance genes (*Pi-2, Pi-54*) along with an additional blight resistance gene (*Xa38*) (Arunakumari et al., 2016; Chukwu et al., 2019; Varshney et al., 2021). Similarly, “Pusa Basmati 1” has been enhanced by stacking multiple blast and blight resistance loci (Varshney et al., 2021). In wheat, MAB has improved resistance to rust diseases through incorporating genes such as *Yr40/Lr57, Lr58*, and *Lr34* into widely adapted cultivars including “Jagger,” “Overley,” and “HUW510” (Luo et al., 2023; Miedaner and Korzun, 2012; Varshney et al., 2021). Comparable advances have occurred in barley through the deployment of powdery mildew and yellow mosaic virus resistance genes (*mlo*, *Pch1*, *rym4/rym5*), and in soybean through the development of cultivars resistant to multiple soybean cyst nematode races (Varshney et al., 2021). Resistance QTLs have also been introgressed into groundnuts to improve rust resistance and yield, while chickpea lines such as C 214 have been strengthened for resistance to both wilt and blight (Varshney et al., 2021).

### 5.2 CRISPR and genome editing

CRISPR/Cas9-mediated genome editing is a groundbreaking advancement in molecular biology, enabling precise alterations of genetic material by exploiting a bacterial immune defense mechanism (Cui et al., 2017). The system uses the Cas9 enzyme, guided by a customizable RNA molecule, to induce double-stranded breaks (DSB) at specific DNA sites, typically recognized by the NGG protospacer adjacent motif (Jinek et al., 2012). These breaks are then repaired through cellular mechanisms like non-homologous end joining (NHEJ) or homology-directed repair (HDR), leading to targeted insertions or deletions. The *Streptococcus* pyogenes Cas9 is the most widely used variant, offering a powerful and versatile tool for genome editing in diverse organisms (Gantz and Akbari, 2018).

#### 5.2.1 Genome editing

Genome editing, also known as gene editing, involves the addition, removal, or replacement of DNA bases in a target sequence to alter gene function using the cell’s natural mechanisms (Bhuyan et al., 2023; Bortesi and Fischer, 2015). CRISPR/Cas9 is the most recent and efficient tool used to develop insect-resistant cultivars by knocking out genes or introducing mutations to suppress harmful gene products (Gao, 2021). For example, it has been used in *Solanum pimpinellifolium* to target loci affecting yield and efficiency (Agustin et al., 2018), and in *Plutella xylostella* to increase resistance to cry1Ac by knocking *out PxABCC2* and *PxABCC3* (Guo et al., 2019). Since many polyphagous insects identify host plants using volatiles, gustatory signals, and visual or tactile cues (Larsson et al., 2004), genome editing of plant volatile profiles may offer a promising pest management strategy.

#### 5.2.2 Mechanism of CRISPR/Cas9 system in genome editing

The CRISPR/Cas9 system, adapted from a bacterial immune response, has become a versatile genome editing tool with well-characterized mechanisms (Hsu et al., 2014; Sander and Joung, 2014). The Cas9-gRNA complex targets DNA sequences adjacent to a PAM (NGG), enabling site-specific cleavage via its HNH and RuvC domains (Jiang et al., 2015). This DSB is repaired by either NHEJ, which often causes indels, or HDR, which enables precise gene modifications such as knockouts or knock-ins. Catalytically inactive Cas9 (dCas9) variants, which bind DNA without cleavage, have expanded CRISPR applications to include gene regulation, epigenetic editing, and genome labeling (Doudna and Charpentier, 2014). Moreover, innovations like the double-nicking strategy have improved on-target specificity and reduced off-target effects, significantly broadening the utility of CRISPR/Cas9 for functional genomics and therapeutic development (Doudna and Charpentier, 2014; Ochiai et al., 2015).

#### 5.2.3 Genetic engineering using CRISPR/Cas9 in insects

The CRISPR/Cas9 system has been successfully used to induce precise mutations in various insect models, demonstrating its broad utility in functional genomics. In *Drosophila melanogaster*, sgRNAs targeting *yellow* and *white* genes achieved up to 88% mutagenesis efficiency in the G0 generation, aided by homologous recombination and high-resolution melting analysis for indel detection (Gratz et al., 2013). Stable fly strains expressing Cas9 under a nanos promoter and sgRNA under a U6 promoter further enhanced this approach (Kondo and Ueda, 2013). In *Aedes aegypti*, CRISPR/Cas9 was first used to modify the *ECFP* gene with a 5.5% knockout rate (Dong et al., 2015), and later enabled multiplex targeting of six genes, including *kmo* and *nix* (Basu et al., 2015). Similarly, in *Bombyx mori*, targeting the *BmBLOS2* gene with sgRNAs and Cas9 mRNA led to a 95% mutation rate and large deletions when co-injected, highlighting the system’s high efficiency in silkworms (Wang et al., 2013). CRISPR/Cas9 has also been employed to investigate the genetic basis and evolutionary dynamics of insect resistance, enabling prediction and validation of resistance mechanisms. In *H. armigera* and *Helicoverpa punctigera*, resistance to the *Bt* toxin Cry2Ab has been associated with loss of function mutations in the ABC transporter gene *ABCA2*. To confirm this relationship, *HaABCA2* knock-out strains were created in *H. armigera* using CRISPR/Cas9. These knock-out strains showed high levels of resistance, demonstrating that *HaABCA2* is critical for mediating Cry2Ab and Cry2Aa toxicity in *H. armigera* (Wang et al., 2017).

#### 5.2.4 Current global regulatory landscape for plant genome editing

The regulatory landscape for genome-edited crops varies considerably across countries, reflecting rapid technological advances and divergent policy frameworks. Nations such as the United States, Argentina, Brazil, and Japan exempt certain genome-edited plants, particularly those developed through SDN-1 or SDN-2 approaches without the introduction of foreign DNA, from strict GMO regulations. In contrast, the European Union and New Zealand regulate all genome-edited organisms under existing GMO legislation (Schmidt et al., 2020; Sprink et al., 2022; Wolt et al., 2015; Wasmer, 2019). India has adopted a tiered regulatory system that exempts SDN-1 and SDN-2 products lacking exogenous DNA while maintaining oversight for SDN-3 modifications (Menz et al., 2020; Prasad and Chimata, 2023). Meanwhile, countries such as China and several African nations are actively developing comprehensive regulatory guidelines, and others including the UK, Norway, and Switzerland are considering reforms to streamline the approval process. Historically, the European Union regulated all genome-edited organisms under GMO legislation. In December 2025, the Council and Parliament reached a provisional agreement on new genomic techniques (NGTs) that aims to enhance competitiveness, strengthen food security, and promote sustainability in the agri-food sector, while maintaining safety standards. The current legislation on CRISPR/Cas is also being reformed in the EU. Under this framework, plants modified using NGTs that are equivalent to conventional varieties may follow a simplified regulatory process, whereas more complex modifications remain subject to GMO rules (Council of the European Union, 2025). Overall, global trends suggest a gradual shift toward more flexible, risk-proportionate regulatory models designed to balance innovation and biosafety consideration (Schmidt et al., 2020; Sprink et al., 2022).

### 5.3 Multi-omics approaches

Multi-omics approaches integrate high-throughput genomics, transcriptomics, proteomics, metabolomics, ionomics, and interactomics to provide a comprehensive view of plant defense mechanisms against insect pests and pathogens. By capturing gene expression, protein activity, metabolite accumulation, nutrient dynamics, and molecular interactions, multi-omics enables the dissection of resistance traits across multiple biological scales (Gothe et al., 2024; Kumaraswamy and Huang, 2024; Varadharajan et al., 2025). Genomics and transcriptomics help identify resistance genes and regulatory networks, whereas proteomics and metabolomics uncover defense-related proteins and secondary metabolites. Ionomics and interactomics further reveal nutrient balance, signaling interactions, and molecular networks that shape defense responses. These approaches have proven effective in unraveling complex traits involving physical barriers, biochemical pathways, and molecular signaling (Alhousari and Greger, 2018; Jing et al., 2024). For example, in *Nicotiana benthamiana*, integrated metabolomics and transcriptomics identified glandular-trichome O-acyl sugars contributing to whitefly resistance (Feng et al., 2021), while combined transcriptomic and metabolomic profiling in wheat revealed defense and stress-response pathways activated during aphid infestation (Zhang et al., 2020b). Multi-omics has also demonstrated that silicon-mediated resistance relies on both mechanical reinforcement and activation of plant defense pathways in silicon-accumulating and non-accumulating species (Alhousari and Greger, 2018). Integrated datasets have additionally identified key signaling proteins and secondary metabolites governing direct and indirect plant defenses (Zhao et al., 2020).

Multi-omics has similarly advanced understanding of plant-pathogen interactions by revealing host immunity components, pathogen virulence factors, and SAR (David et al., 2020). In *Brassica* species, multi-omics has elucidated gene networks and metabolite shifts associated with resistance to bacterial and fungal pathogens, including the discovery of QRLs and calcium-signaling pathways (Shaw et al., 2021). Coupling these datasets with high-throughput phenotyping and computational modeling enables the identification of biomarkers, regulatory hubs, and metabolites crucial for resistance (Gothe et al., 2024; Mahmood et al., 2022);. These discoveries directly support targeted breeding, genomic selection, and engineering of crops with enhanced resilience (Kumari et al., 2022; Shaw et al., 2021). Despite its promise, multi-omics research faces challenges, including the integration of diverse and heterogeneous datasets, sensitivity to environmental and genotypic variation, and difficulties translating laboratory findings to field settings (Barah and Bones, 2014; Crandall et al., 2020; Varadharajan et al., 2025). Functional validation remains essential to avoid overinterpreting correlations. Regulatory and biosafety considerations also limit deployment of engineered plants or microbes derived from omics-based strategies (Hoffmann et al., 2023). Nonetheless, advances in synthetic biology, genome editing, and microbiome engineering when paired with multi-omics datasets are poised to overcome many of these limitations. Improvements in machine learning (ML), data integration, and predictive modeling will further strengthen the role of multi-omics in resistance research and crop improvement (Wekesa and Kimwele, 2023). Overall, multi-omics has transformed the study of plant defense by revealing molecular complexity and enabling innovative breeding and biotechnological strategies for sustainable pest and disease management (Bisht et al., 2023; Shaw et al., 2021).

### 5.4 RNA interference

**Figure 5:**
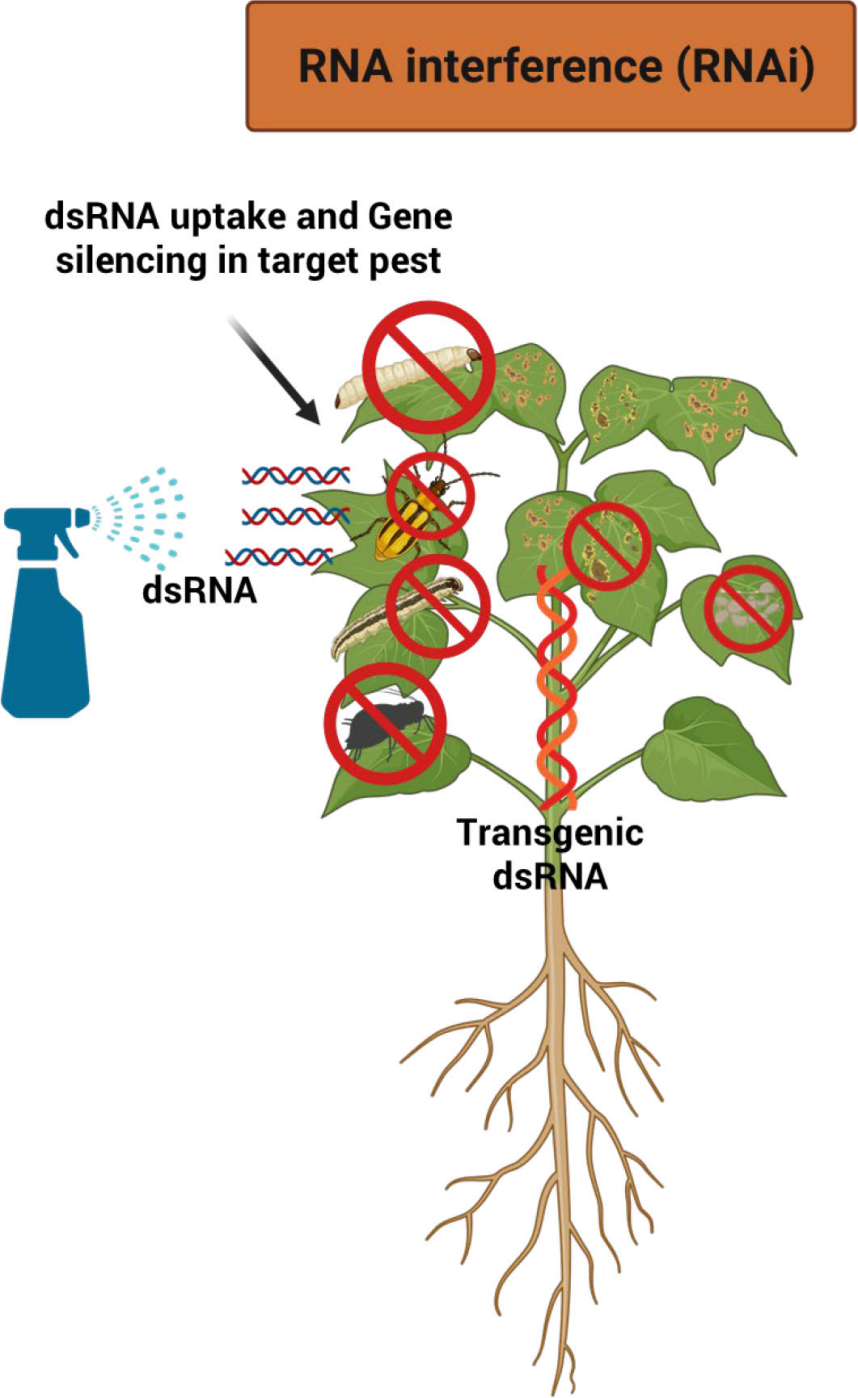
Overview of RNA interference (RNAi)-mediated crop protection.

RNAi, also known as post-transcriptional gene silencing (PTGS), is a sequence-specific and environmentally friendly mechanism that suppresses gene expression through double-stranded RNA (dsRNA) triggers (Fire et al., 1998; Kamthan et al., 2015). By exploiting this naturally occurring regulatory pathway, RNAi can target essential insect genes with high precision, resulting in impaired development, reduced fecundity, or mortality (Jaba and Sharma, 2017; Li et al., 2023a) (**Figure 5**). Following uptake, dsRNA is processed by the RNAi machinery into small interfering RNAs (siRNAs) of approximately 20 nucleotides, which guide Argonaute proteins within the RNA-induced silencing complex (RISC) to degrade complementary target mRNAs, thereby preventing translation of essential proteins (Scott et al., 2013).

RNAi-based plant protection has been successfully applied in several crop-pest systems. In maize, silencing *V-ATPase*, *dvvgr*, and *dvbol* genes enhanced resistance to Western corn rootworm (Fishilevich et al., 2016; Niu et al., 2017; Li et al., 2015). In rice, suppression of *APN1*, *APN2*, and acetylcholinesterase reduced susceptibility to striped stem borer and yellow stem borer (Qiu et al., 2017; Kola et al., 2019). In tobacco and tomato, silencing *HaCHI* or *HACHI* genes disrupted *H. armigera* development (Mamta et al., 2016; Mamta and Rajam, 2017), while targeting phenolic glucoside malonyltransferase increased resistance to whiteflies (Xia et al., 2021). Similarly, suppression of *MyCP* gene in *Arabidopsis* reduced aphid fecundity (Bhatia and Bhattacharya, 2018). Advances in nano-carrier-based dsRNA delivery have further improved RNAi efficiency, resulting in high aphid mortality in soybean (Yan et al., 2020). Transgenic plants expressing dsRNA, also known as plant-incorporated protectants (PIPs), have demonstrated robust and heritable insect resistance. Notably, the first RNAi-based maize approved by the U.S. Environmental Protection Agency targets the *Snf7* gene in *Diabrotica* spp., combined with Cry proteins and glyphosate tolerance (Ramaseshadri et al., 2013; Fishilevich et al., 2016; U.S. Environmental Protection Agency, 2017). Collectively, these studies highlight RNAi as a promising, species-specific, and sustainable strategy for developing insect-resistant crop (Meng et al., 2026; Dong et al., 2024).

Beyond insect management, RNAi also plays a critical role in antiviral plant defense (Calil and Fontes, 2016; Lopez-Gomollon and Baulcombe, 2022). Transgenic rice and other crops engineered for RNAi-mediated resistance have shown substantial resistance to tenuiviruses, reoviruses, and geminiviruses (Sasaya et al., 2014; Zhang et al., 2005). Through host-induced gene silencing (HIGS), RNAi has also proven effective against bacterial and fungal pathogens (Bilir et al., 2022; Bocos-Asenjo et al., 2022). Additionally, host-delivered RNAi represents a promising strategy for controlling plant-parasitic nematodes by disrupting genes essential for their development and pathogenesis (Banerjee et al., 2017; Lilley et al., 2007). Despite its considerable potential, large-scale implementation of RNAi technologies remains constrained by challenges related to dsRNA stability, delivery efficiency, and production costs (Meng et al., 2026; Scott et al., 2013). Continued advances in formulation, delivery platforms, and cost-effective dsRNA synthesis will be essential for translating RNAi into widely adopted crop protection strategies.

### 5.5 Microbiome-assisted resistance

Plant-associated microbial communities play a critical role in mediating resistance to insect herbivores and pathogens through a range of direct and indirect mechanisms (Liu et al., 2024; Sanchez-Mahecha et al., 2022). Beneficial microbes can induce systemic resistance, enhance the production of defensive secondary metabolites, and reshape the plant microbiome to create environments less favorable to pests (Aravinthraju et al., 2024; Liu et al., 2024). Soil and rhizosphere microbiomes contribute substantially to these defenses by priming immune responses, improving nutrient availability, and suppressing pest populations (Blundell et al., 2019; Harmsen et al., 2024; Qadri et al., 2020). Microbial signaling molecules, such as N-acyl homoserine lactones, further modulate plant-insect-microbe interactions in ways that depend on environmental context and host genotype (Sanchez-Mahecha et al., 2022). In parallel, insect gut microbiomes influence host susceptibility, offering new opportunities for microbiome-targeted pest control strategies (Zhang et al., 2023). Advances in microbiome engineering seek to enrich beneficial rhizosphere communities or introduce synthetic microbial consortia to enhance immunity and resilience against diverse insect pests (Liu et al., 2024). Rhizosphere microbiomes also play a central role in resistance to soil-borne and foliar pathogens by fostering beneficial taxa that suppress pathogens and activate plant immune pathways (Ali et al., 2023; Bakker et al., 2020). Beneficial microbes commonly induce systemic resistance (ISR), priming plant defenses through SA, JA, and ET signaling pathways (Ali et al., 2023; Bakker and Berendsen, 2021). Disease-suppressive soils highlight how specific root-associated microbiota can confer transferable resistance to other soils, providing a foundation for microbiome-based disease management (Bakker et al., 2020; Harmsen et al., 2024).

Synthetic biology approaches including the development of synthetic microbial communities (SynComs) enable precise manipulation of the microbiome to strengthen plant health and disease resistance (Ruiz et al., 2025). These interactions are strongly shaped by core microbiome members and their ecological networks (Roy et al., 2024). Because microbiome-mediated resistance often acts through SA, JA, and ET pathways, these interactions can simultaneously enhance resistance to both pathogens and insects (Ali et al., 2023; Schenk et al., 2008). Recent studies indicate that breeding for microbiome-compatible plant genotypes or designing SynComs could help establish protective microbiota that reinforce these defense pathways (Kusstatscher et al., 2021). Overall, microbiome-assisted resistance offers a sustainable strategy for reducing pesticide reliance and improving crop resilience by harnessing beneficial interactions between plants and their microbial partners (Ali et al., 2023; Kumar et al., 2023).

### 5.6 Synthetic biology for resistance

Genetic transformation is a transformative tool in modern plant breeding, enabling precise modification of plant genomes and offering faster, more targeted improvements than traditional methods. Classified among new breeding techniques (NBTs), genetic engineering allows insertion, deletion, or modification of specific genes, dramatically accelerating the development of crops with enhanced resistance to pests, diseases, and environmental stresses, as well as improved nutritional quality and yield (Robinson, 2001; Schaart et al., 2016; Chou et al., 2020). By facilitating gene transfer across species barriers, these approaches expand the range of traits accessible for crop improvement beyond the limitations of conventional breeding (Hamdan and Tan, 2024; Zhang et al., 2024). Wide crosses involving distantly related species often require genetic transformation to overcome reproductive barriers and introduce desirable traits (Tarafdar et al., 2014). Through the stable integration of foreign DNA into plant genomes, transgenic plants can be produced using molecular biology, tissue culture, and gene transfer tools without the need for fertilization (Alves et al., 1999; Anjanappa and Gruissem, 2021; Babaoglu et al., 2000). Synthetic biology uses engineering-based design cycles to modify plant genetic circuits for improved crop traits and productivity. Combined with tools such as gene editing, multi-omics, and artificial intelligence (AI), it enables the development of crops with enhanced efficiency, resilience, and nutrient use. AI-powered approaches also support the creation of SMART crops, though practical challenges remain for widespread application (Zhang et al., 2024).

### 5.7 Exploring novel sources of resistance

Novel resistance sources against insect pests and diseases are being explored using natural compounds, biocontrol agents, and advanced breeding strategies (Douglas, 2018; Ramaroson et al., 2022; War et al., 2012). These approaches support reduced pesticide use and sustainable agriculture (Kumari et al., 2022; Souto et al., 2021; Zehra et al., 2021). Plants utilize a range of biochemical defense strategies against insect and pathogen attacks, producing compounds that can be constitutive or induced in response to damage (War et al., 2012). These include over 25,000 terpenoid structures, as well as phenylpropanoids and flavonoids, which act as barriers, toxins, or signaling agents in stress responses (Ramaroson et al., 2022). VOCs also contribute by attracting natural enemies of herbivores, with compounds like 3-pentanol and 2-butanone shown to induce resistance and increase populations of beneficial insects (Song and Ryu, 2013). Additionally, plant-derived pesticides such as terpenes, flavonoids, and alkaloids present eco-friendly alternatives to synthetic chemicals due to their diverse modes of action and biodegradability (Souto et al., 2021).

Biocontrol agents (BCAs), including fungi, bacteria, and viruses, are increasingly viewed as eco-friendly alternatives to chemical pesticides for managing plant pests and diseases (Egamberdieva et al., 2023; Zehra et al., 2021). They suppress pathogens through antibiosis, competition, mycoparasitism, enzyme production, and by triggering plant defense responses (Egamberdieva et al., 2023; Harman, 2006; Zehra et al., 2021). *Trichoderma* spp. and *Bacillus subtilis* are well-known BCAs that promote plant health by reducing pathogen load, improving stress tolerance, and enhancing nutrient uptake (Harman, 2006; Hashem et al., 2019; Luo et al., 2023). Some agents, such as hrp mutant bacteria, induce basal resistance by colonizing plant tissues (Hanemian et al., 2013). BCAs also stimulate ISR and SAR, which provide broad-spectrum protection against both pathogens and insect pests (Harman, 2006; Song and Ryu, 2013; War et al., 2012).

Modern breeding and genetic engineering provide effective strategies for enhancing plant resistance. Conventional breeding has developed insect-resistant cultivars and identified resistance traits in wild relatives. Molecular tools such as MAS, gene pyramiding, RNAi, and genome editing expedite resistance breeding (Kumari et al., 2022). Genetic engineering enables the transfer of insecticidal genes like *Bt*, protease inhibitors, and lectins into crops (Kumari et al., 2022). Genome editing technologies such as CRISPR/Cas9 offer precision in modifying susceptibility or resistance genes, enhancing disease resistance (Kumari et al., 2022; Manzoor et al., 2024; Purnhagen et al., 2021). These tools can also target pathogen genomes directly (Deng et al., 2020; Manzoor et al., 2024). For instance, *Triticum monococcum* shows resistance to take-all disease, and the oat avenacin biosynthetic pathway presents opportunities to engineer similar defenses in wheat (Palma‐Guerrero et al., 2021). Additionally, increasing crop diversity through intercropping and rotation can suppress pests and diseases by altering their ecological dynamics (He et al., 2019).

### 5.8 Impact of artificial intelligence on crop breeding

AI is increasingly transforming plant science and accelerating trait selection in crop improvement, particularly for biotic stress resistance, yield potential, and climate resilience (Farooq et al., 2024). One longstanding bottleneck in conventional breeding has been the accurate and high-throughput measurement of plant traits (i.e., phenotyping). Recent advances demonstrate how deep learning (DL) and ML algorithms can substantially improve phenotypic analysis. For example, a recent study showed that DL/ML-based image analysis precisely quantified insect damage on grapevine leaves and integrated these AI-derived phenotypes with genomic data to accurately predict resistance traits (Gan et al., 2025). This work highlights the power of AI in plant phenomics and genomic selection, facilitating genomic breeding of pest-resistant grapevine.

Beyond phenotyping, AI is reshaping how plant geneticists and breeders interpret genetic information. Rather than relying solely on marker-trait associations, AI-driven approaches can predict key genes and infer their functional impacts, thereby enhancing precision, efficiency, and sustainability in breeding programs. AI has also emerged as a transformative tool in crop protection strategies (Madhuri et al., 2025). It accelerates the discovery, design, and optimization of agricultural synthetic bioactives, supporting the development of bio-based, eco-friendly alternatives to conventional pesticides, including biopesticides, improved fertilizers, and stress-resilient crop traits. Looking ahead, it will be critical to establish an intelligent breeding loop integrating AI-based prediction, gene editing, and robotic execution; to advance agricultural large language models (Agri-LLMs) for broader and more inclusive applications; to develop sustainable breeding evaluation systems; and to empower smallholder farmers through edge computing technologies (Cai et al., 2025). A recent perspective in *Nature* (Li et al., 2025a) proposed an “AI-assisted crop design” paradigm, in which AI systems analyze multimodal datasets, including genomic, phenotypic, and environmental information, to generate optimized breeding strategies tailored to specific breeder objectives, such as enhanced yield or improved stress resistance.

Despite these breakthroughs and promising perspectives, challenges remain. The “black-box” nature of some DL models limits biological interpretability, making it difficult for breeders and biologists to fully understand the mechanistic basis of predictions. As AI continues to integrate with synthetic biology, the development of ideal crop varieties with unprecedented speed and precision appears increasingly feasible, but careful validation, transparency, and interdisciplinary collaboration will be essential to realize this potential.

## 6 Benefits of plant resistance

### 6.1 Reduced reliance on pesticides

Plant resistance plays a central role in reducing reliance on chemical pesticides and advancing sustainable agriculture by capitalizing on intrinsic genetic traits that deter, withstand, or suppress pest and pathogen attack (Ali et al., 2023; Gossen and McDonald, 2019; Johnson et al., 2017; VanDoorn and De Vos, 2013). These built-in defenses reduce production costs, preserve beneficial organisms, and align with consumer expectations for environmentally responsible practices by lowering pesticide residues in food and the environment (Mitchell et al., 2016). Plant resistance also slows the evolution of pesticide resistance in pest populations and strengthens IPM programs. In the context of increasingly stringent regulations, limited chemical options, and market pressures, resistance breeding remains a cornerstone strategy for reducing synthetic inputs across agricultural systems (Johnson et al., 2017; VanDoorn and De Vos, 2013).

### 6.2 Self-defense and economic management

Plants deploy both constitutive defenses (e.g., thick cuticles, trichomes) and induced defenses (e.g., secondary metabolites, proteinase inhibitors) to mitigate damage (Kumaraswamy et al., 2024a, 2025). Induced responses rely on signaling pathways that activate the synthesis of defensive compounds such as phenolics and alkaloids. R genes allow specific pathogen recognition and can trigger hypersensitive cell death to confine infections. For insects, resistance may arise through antibiosis, antixenosis, or tolerance, reducing pest growth, deterring feeding, or mitigating damage without impairing plant fitness (Bailey-Serres et al., 2019). Economically, resistance offers substantial advantages by lowering dependence on chemical control, reducing input and labor costs, and minimizing the risk of pest adaptation (Zhou et al., 2024). Resistant cultivars are particularly beneficial in resource-limited regions where fewer pesticide applications translate into significant cost savings. They also help stabilize yields under pest pressure and maintain natural enemy populations, improving the durability and profitability of broader pest management strategies.

### 6.3 Sustainable agriculture

Deploying resistant cultivars reduces dependence on synthetic pesticides, thereby lowering environmental contamination, protecting non-target organisms, and supporting consumer demand for residue-free food (Zhou et al., 2024). Resistant plants sustain yields under pest and pathogen pressure, especially benefiting smallholder farmers with limited access to chemical inputs. This stability enhances food security and lowers production costs by reducing the need for pesticide use and labor. As a core component of IPM, plant resistance complements other strategies and helps delay pest adaptation, preserving resistance durability (Kumaraswamy et al., 2024a). In the context of climate change, resistant varieties are vital for ensuring crop resilience against emerging biotic stresses.

## 7 Challenges, limitations, & future directions

Despite major advances in understanding plant defense pathways, several challenges constrain the effective use of resistance traits in crop protection (Huang, 2011). Polygenic resistance remains difficult to dissect and breed due to its complex genetic architecture and sensitivity to environmental conditions. Race-specific resistance can be rapidly overcome by pathogen or insect evolution, and resistance–yield trade-offs complicate deployment in breeding programs. Moreover, omics-based studies may lack sufficient resolution to reveal subtle regulatory interactions underlying durable resistance. Future progress will depend on integrating multi-omics frameworks to model defense networks, applying precise genome-editing tools such as CRISPR/Cas for resistance stacking, and exploring broad-spectrum strategies including defense priming, RNAi, and microbiome-mediated resistance. Combining molecular, ecological, and system-level approaches will be critical for building sustainable and climate-resilient crop protection.

### 7.1 Durability *vs*. insect/pathogen evolution

Resistance durability, its sustained effectiveness across environments and overtime, is continually challenged by the adaptive evolution of pests and pathogens. Many pathogens and insect herbivores evolve rapidly through mutation, recombination, or horizontal gene transfer, enabling them to bypass plant defenses. Examples include rust fungi and aphid populations that repeatedly overcome widely deployed R genes, leading to recurrent cycles of resistance breakdown. Herbivores may also evolve enhanced detoxification capacity or altered feeding behavior to circumvent plant chemical defenses. Enhancing durability requires strategies that reduce the selection pressure imposed by single resistance mechanisms. These include pyramiding multiple R genes with diverse modes of action (Zhang et al., 2022), deploying cultivar mixtures or rotations with distinct resistance backgrounds, and integrating biological control. Incorporating inducible defenses or traits that indirectly reduce pest fitness, alongside advanced tools such as RNAi and CRISPR/Cas, can further help maintain long-term resistance effectiveness.

### 7.2 Trade-offs: balancing resistance with growth/yield (fitness cost of R-genes)

The deployment of R genes in plants is a key breeding strategy to reduce losses from pathogens and pests, but it often involves fitness costs due to trade-offs between enhanced defense and important agronomic traits like growth and yield (Zhang et al., 2022). R genes encode receptors that detect specific pathogen effectors, triggering defense responses such as HR, ROS production, and defense metabolite synthesis. While these responses limit pathogen spread, they also divert metabolic resources from growth and reproduction. The fitness cost of R genes is not universal but depends heavily on the environmental context. While plants carrying certain R genes may exhibit reduced biomass or yield in pathogen-free conditions, they often outperform susceptible plants under disease pressure, compensating for these costs. This highlights the need to consider environment-specific fitness when assessing R gene value. Modern breeding strategies focus on optimizing resistance by stacking R genes with varied activation thresholds, using tissue-specific or inducible promoters, and incorporating QRLs for partial, broad-spectrum defense with lower costs. Advances in genome editing further enable precise modifications of R genes or their regulators to reduce negative effects on growth.

### 7.3 Limited resistance to certain stresses in crops

Despite advances in breeding and biotechnology, many crops still show limited resistance to specific biotic and abiotic stresses, which hinder sustainable agriculture and food security. This is largely due to the narrow genetic base of cultivated crops, often lacking resistance alleles because intensive selection for yield has reduced genetic diversity. Complex stresses such as drought, heat, and diseases involving multiple pathogens require polygenic resistance controlled by many small-effect genes influenced by the environment, making breeding difficult and slowing the development of stable, resilient cultivars. Consequently, crops may have only partial tolerance rather than durable resistance. Additionally, pests and pathogens evolve faster than crops can adapt, with specialist herbivores bypassing defenses and pathogens overcoming race-specific resistance genes through mutation, creating ongoing challenges in maintaining effective resistance.

In some cases, no wild relatives or landraces with resistance genes exist for certain stresses, as seen in banana for Fusarium wilt Tropical Race 4 and citrus for Huanglongbing, which limits breeding options. Additionally, some crops have weak or incomplete defense pathways, such as low osmoprotectant accumulation under salt stress or poor induction of ROS scavenging enzymes under heat, increasing their susceptibility. To overcome these challenges, expanding genetic diversity through pre-breeding, introgression from wild species, or genome editing is necessary, along with a deeper understanding of stress-specific resistance mechanisms. Combining molecular tools, high-throughput phenotyping, and ecological management offers an integrated approach to build crop resilience against current and future stresses.

## 8 Conclusion

Plant resistance to insects and diseases is a cornerstone of sustainable agriculture and a key component of IPM. Evidence indicates that resistance is not governed by a single process, but by a complex and dynamic interplay of constitutive and inducible defenses, genetic determinants, biochemical pathways, and ecological interactions (Huang and Huang, 2023). Structural barriers, secondary metabolites, innate immune responses, and sophisticated signaling networks together enable plants to recognize biotic threats and activate defenses that limit insect damage and disease development. Recent advances in molecular biology and biotechnology have greatly expanded our understanding of resistance mechanisms. The discovery of resistance genes, TFs, QTL, and defense-associated metabolites has yielded important targets for crop improvement. Modern approaches, including RNAi, CRISPR/Cas9-mediated gene editing, and MAB, provide unprecedented precision for developing resistant cultivars, while multi-omics technologies are elucidating the regulatory networks underlying durable resistance. Critically, HPR reduces reliance on chemical pesticides, thereby lowering environmental contamination, preserving beneficial organisms, and minimizing risks to human health, while also helping to manage evolving pest populations. Future research should prioritize the integration of resistance traits with agronomic performance, yield stability, and climate resilience. Exploiting natural variation in wild relatives, pyramiding complementary resistance mechanisms, and incorporating ecological interactions will be essential for long-term sustainability. Strengthening plant resistance through interdisciplinary approaches will be central to building resilient cropping systems, conserving biodiversity, and ensuring global food security amid increasing biotic pressures and environmental change.
